# Supplementary material for: A scoping review of measurement of violence against women and disability
Source: PLoS One. 2022 Jan 31;17(1):e0263020. doi: 10.1371/journal.pone.0263020 (PMC8803172; doi:10.1371/journal.pone.0263020)
Supplement: S2 Appendix — (DOCX) [file pone.0263020.s002.docx]

**Appendix 2: Data extraction variables:**

***Study characteristics:***

- Study setting (country, income-level, WHO-region);
- Study design;
- Research questions;
- Study setting (community, institution);
- Data collection method(s): survey administration, type of measurement instrument;
- Comparisons included: women with disability vs. women without disability; men with disability vs. women with disability; no comparison;
- Sampling approach (sample size, inclusion and exclusion criteria, recruitment methods); and
- Sample characteristics (only women included; only persons with disability included).

***Measurement of violence:***

- Type(s) of violence included;
- Context of violence (relationship, institution);
- Perpetrator(s);
- Scale/ items used to assess violence;
- Time frame; and
- Severity of violence measured (yes/ no).

***Measurement of disability:***

- Type(s) of disability(ies) included;
- Definition of disability utilized;
- Scale/ items used to assess disability;
- Time frame of disability included (yes/ no); and
- Severity of disability measured (yes/ no).
