# Supplementary material for: A scoping review of measurement of violence against women and disability
Source: PLoS One. 2022 Jan 31;17(1):e0263020. doi: 10.1371/journal.pone.0263020 (PMC8803172; doi:10.1371/journal.pone.0263020)
Supplement: Attachment — Submitted filename: Response to reviewers.docx [file pone.0263020.s003.docx]

**Authors’ response to reviewers**

**Manuscript title:** A scoping review of measurement of violence against women and disability

To the Editors, PLoS One

Thank you for the recognition of the contribution of our manuscript, “A scoping review of measurement of violence against women and disability.” In response to the reviewer’s comments, some changes have been made to the manuscript. We appreciate the reviewer’s positive comments and feel that our responses to these helpful suggestions helped improve this manuscript. The reviewers’ comments, as well as journal requirements listed, are addressed point-by-point in turn below.

**Editorial comments:**

1. Please ensure that your manuscript meets PLOS ONE's style requirements

We have formatted the manuscript according to these style requirements.

2. Grant information: We have removed the funding information from the manuscript. The funding information that is now in the online submission form reads as follows:

“This work was funded under the UNWomen-WHO Joint Programme on Violence against Women Data, funded by the Foreign, Commonwealth and Development Office. The funders had no role in study design, data collection and analysis, decision to publish, or preparation of the manuscript.”

**Reviewer comments:**

1. A general definition of disability or how authors defined disability early on in the paper could be included.

We have added the following definition on Page 7:

Disability is defined as “the interaction between individuals with a health condition…with personal and environmental factors including negative attitudes, inaccessible transportation and public buildings, and limited social support,” [18]; the Convention on the Rights of Persons with Disabilities also emphasizes social participation, such that “disability results from the interaction between persons with impairments and attitudinal and environmental barriers that hinders their full and effective participation in society on an equal basis with others” [19].

2. On page 13 studies were excluded if they focused only on common mental health disorders—rationale could be provided as to why these were excluded.

We have added the following rationale.

Common mental disorders were excluded as there is a robust evidence-base on VAW and common mental disorders. This evidence-base includes several systematic reviews and meta-analyses [33-35], and therefore we focused this review on an area with less well-developed measurement and methodology.

3. The authors cover three bodies of literature and define two of these-disability focused research and intersection of disability and violence on page 10, could also elaborate more on the third-“measurement of disability in the context of research focused on violence against women”.

We have added the following definition on Page 11: “We define measurement of disability in VAW research as research that focuses on questions of prevalence of violence that measure disability as a specific risk factor or variable within study objectives focusing on understanding VAW in a population or specific group.”

4. Page 17 says, 419 selected for full text review and an additional 269 were excluded after that. The count is 150 after exclusion and not 174. The sentence “A final 174 reports or manuscripts met the inclusion criteria” could be deleted from that para—that para is focusing only on peer reviewed literature. The next para could then be rephrased for clarity and the final count can be mentioned in that para.

We have edited as suggested, and the second paragraph now reads as follows:

The grey literature search was conducted separately, and identified 316 reports, of which 5 were selected for inclusion. In addition, 16 Demographic and Health Surveys and 3 reports of national violence against women studies met the inclusion criteria. With the 150 peer-reviewed manuscripts, a final 174 reports or manuscripts met the inclusion criteria.

5. Discussion/conclusion—The authors could elaborate more on how the gap in literature is impacting practice and policies for disabled women with exposures to violence and how addressing this gap is important for practitioners and policy makes

We have added the following text to the Discussion section: “Our findings indicate limitations in measurement and evidence-base, which impact policy-makers and programmers given the overall prevalence of violence against women with disabilities is unknown, which limits development and implementation of effective policies and support services. In addition, the lack of evidence concerning how different types of disabilities operate as risk factors compared to others, and can create different barriers and enables for women who experience violence seeking support, hampers effective programming tailored to specific needs.”
